# Social Media and Adolescents’ Well-Being

**DOI:** 10.3390/healthcare11162297

**Published:** 2023-08-15

**Authors:** Sijia Guo, Chau-Kiu Cheung

**Affiliations:** 1 College of Public Administration and Humanities, Dalian Maritime University, Dalian 116026, China; sijiaguo@dlmu.edu.cn; 2 Department of Social and Behavioural Sciences, City University of Hong Kong, Hong Kong 518000, China

The call for articles for the Special Issue of *Healthcare*, entitled “Social Media and Adolescents’ Well-Being”, was proposed at the beginning of 2021 as part of multidisciplinary efforts to understand the complex interactions between social media usage and adolescents’ well-being.

Adolescence, as a transitional period, exerts a remarkable influence on individuals’ future mental status and personal functioning. As such, extant research has explored adolescents’ behaviors, interpersonal relationships, emotional status, social functioning, social adaptation, and so on [1,2,3]. Nowadays, embedded within a society with unprecedented changes, adolescents deal with numerous challenges undermining or promoting their personal well-being, such as complex interpersonal relationships. One such notable circumstance is the prevalence of social media. Currently, social media usage is an ever-increasing phenomenon in adolescents’ daily lives, raising public concern about its effects on adolescents’ well-being. Reports from the U.S. indicated that the most popular social media platforms among teenagers were YouTube, Instagram, and Snapchat, and over 93% of teenagers ranging from 12 to 17 years old were connected to social media [4]. Likewise, an annual report from China showed that approximately 183 million teenagers used the Internet and social media platforms to communicate with others; watching short videos and studying were adolescents’ popular online activities [5]. Accordingly, the increasing prevalence of social media could be observed. Therewith, growing research from different disciplines sheds light on the effects and results generated by social media on adolescents’ well-being.

Social media count as a ‘double-edged sword.’ On the one hand, some studies focused on the detrimental impacts generated by social media usage. Specifically, excessive frequency or time spent on social media intensifies depression or anxiety, as adolescents fear loss, which compels them to check and respond to their friends’ comments [6,7,8]. In addition, with increasing involvement with social media, the possibilities increase for adolescents to form problematic social media usage habits, such as addiction to social media, which can lead to harmful results, including cyberbullying, social isolation, sleeping problems, and so on [9,10,11]. Moreover, the diversified social media platforms provide abundant channels for adolescents to gain information from others with whom they are familiar or unfamiliar offline. From this perspective, some studies indicated that social media use could increase the frequency of self-presentation and self-disclosure while decreasing self-esteem and mental health. Yet, on the other hand, some researchers argue for the benefits of social media for adolescents’ well-being. Social media, as a platform, give adolescents more opportunities to access more people, information, and resources that further increase their personal social capital and social support. Meanwhile, some studies also claim that social media create a field for adolescents to express themselves and explore their identities, which benefit adolescents’ identity formation [12,13,14]. In line with the results of the previous studies, the results from studies about the impacts of social media on adolescents’ well-being are conflicting. Thus, it is necessary to continue the research on social media and adolescents’ well-being.

Furthermore, although empirical studies have primarily investigated the differential influence of gender, age, or self-esteem status on social media use and its consequences, some other questions still need exploration and discussion regarding the relationship between social media use and adolescents’ well-being [15,16,17]. For example, the question of whether cultural values or norms influence adolescents’ use orientations, patterns, and consequences is worth exploring. Additionally, the factors of parents, peers, or individuals are worth investigating in the future. Social media have become an important part of adolescents’ lives, so the essential task for us is to lower the potential adverse effects and promote the beneficial effects of social media. Herein, this Special Issue explores the relationship between social media usage and adolescents’ well-being while considering the potential protective or detrimental psychological and social factors that influence the mechanism between social media usage and adolescents’ well-being.
